# Efficient circRNA Detection Using the Processive Reverse Transcriptase uMRT

**DOI:** 10.21769/BioProtoc.5480

**Published:** 2025-10-20

**Authors:** Ruben Warkentin, Anna Marie Pyle

**Affiliations:** 1 Department of Molecular, Cellular and Developmental Biology, Yale University, New Haven, CT, USA; 2 Department of Chemistry, Yale University, New Haven, CT, USA; 3 Howard Hughes Medical Institute, Chevy Chase, MD, USA

**Keywords:** circRNA, UltraMarathonRT, RT-PCR, Sanger sequencing, Nanopore sequencing, Rolling circle amplification

## Abstract

Circular RNAs (circRNAs) are covalently closed RNA molecules known for their increased stability compared to linear RNAs. Synthetic circRNAs are being developed as RNA therapeutics, while natural circRNAs are being investigated for their biological roles in eukaryotes and their potential as disease biomarkers. Consequently, the accurate detection and validation of circRNAs is crucial for advancements in both fundamental RNA research and biotechnological applications. Common methods for circRNA validation involve RT-PCR using divergent primers, followed by sequencing across the circRNA junction. However, most described methods are high-throughput approaches that require time-consuming RNA processing steps, and they are unable to detect highly structured circRNAs. Additionally, methods for low-throughput sequencing of small circRNAs (<150 nt) require cloning prior to sequencing. A simplified protocol for the validation of circRNA sequences irrespective of structure, sequence complexity, and length has not yet been described. In this method, we describe an improved RT-PCR protocol for circRNA detection by using UltraMarathonRT^®^ (uMRT), a highly processive reverse transcriptase. Unlike other reverse transcriptases, uMRT can reverse-transcribe large, structured circRNAs of varying sizes, at ambient temperatures, enabling sequencing of the resulting concatemeric amplicons generated by RT-PCR and other methods. Using this method, we sequenced circRNAs containing highly structured internal ribosome entry sites commonly utilized in synthetic circRNAs, natural circRNAs containing repetitive elements, and small circRNAs, all without the need for cloning. With this new platform, we offer a protocol for the precise detection of nearly any circRNA species.

Key features

• This protocol shortens current methods for circRNA detection and sequencing by sequencing RT-PCR products directly, without the need for cloning or processing the PCR product.

• This protocol describes a simple and cost-effective RT-PCR method for single circRNAs.

• The highly processive UltraMarathonRT (uMRT) functions at ambient conditions, reducing circRNA degradation.

• This protocol enables sequencing of circRNAs that are below 150 nt as well as larger circRNAs.

• This protocol enables the detection of structured and very large circRNAs.

## Graphical overview


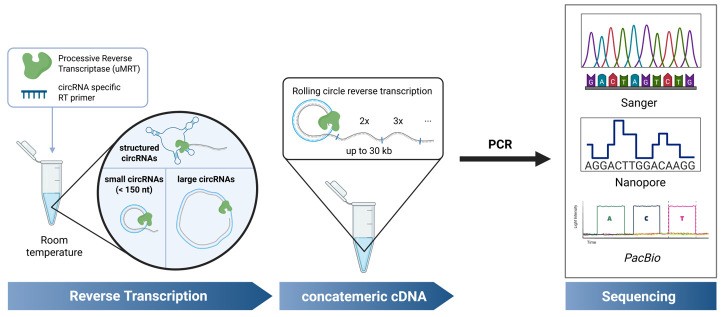


## Background

Circular RNAs (circRNAs) are covalently closed RNA molecules that exhibit increased stability due to their resistance to exonuclease cleavage [1]. circRNAs have been implicated in a variety of biological functions [2], including human diseases such as cancer [3] and neurological disorders [4], where circRNAs have shown potential as biomarkers [5]. Additionally, circRNAs are considered attractive candidates for RNA therapeutics because of their enhanced stability and protein production compared to linear RNAs [6,7]. Given the growing interest in circRNAs for these applications, accurate detection and sequence validation are crucial.

Numerous RT-PCR-based sequencing methods have been described for identifying natural circRNAs in cell extracts [8,9]. These are high-throughput methods designed to detect the circRNA transcriptome by utilizing RNA enrichment strategies, such as ribosomal RNA (rRNA) depletion and RNase R digestion (to eliminate linear RNAs) [10]. However, these approaches are too costly and time-consuming for the verification of individual circRNAs, such as those synthetically made or individual natural circRNAs. Currently, individual circRNAs are sequence-verified through RT-PCR followed by Sanger sequencing across the circRNA junction, or nanopore sequencing [6,7,11,12]. However, these approaches have several notable limitations. For small circRNAs (~100 nt), the resulting RT-PCR product is too short for Sanger and Nanopore sequencing, thus necessitating a cloning step prior to sequencing, as utilized by Du et al. [7]. This additional cloning step increases the time and cost of the verification process. Additionally, RT-PCR is typically performed using retroviral reverse transcriptases (RTs) [13,14], which stall on structured RNAs [15], restricting detection to unstructured circRNAs within a limited size window. While more highly processive RTs have been employed for circRNA detection [16], existing protocols require the use of high temperatures during cDNA synthesis that may shear and degrade RNA circles. Moreover, they have only been used for high-throughput sequencing of cellular circRNAs. Currently, there is no simple protocol for sequencing individual synthetic or cellular circRNA sequences by RT-PCR, irrespective of circRNA structure, sequence complexity, or length.

The method described here enables the straightforward validation of individual circRNA sequences, without the need for enrichment strategies or cloning, by using the highly processive UltraMarathonRT (uMRT) [17,18]. This approach utilizes RT-PCR with uMRT and commercial Nanopore or Sanger sequencing to rapidly and cost-effectively validate small, large, and structured circular RNAs at room temperature. Although this protocol focuses on validation of individual circRNAs, these highly processive RTs could be used for transcriptome-wide RNA sequencing of natural circRNAs or in sequencing approaches that circularize a transcriptome [19]. Applying uMRT to transcriptome-level approaches will require further experiments, but it has the potential to reveal novel circRNAs, including those containing structured regions and other complex elements.

## Materials and reagents

**Reagents**

1. DNA Clean and Concentrator-25 (Zymo Research, catalog number: D4033)

2. uMRT Reverse Transcriptase kit (RNAConnect, catalog number: catalog number: R1002S)

3. Deoxynucleotide (dNTP) solution mix (10 mM each) (NEB, catalog number: N0447S)

4. RNaseOUT^TM^ (Invitrogen^TM^, catalog number: 10777019)

5. CircRNA template or cell extracted RNA

6. RNA loading dye (2×) (NEB, catalog number: B0363S)

7. Reverse transcription primer (should not span circRNA junction)

8. DreamTaq PCR Master Mix (Thermo Fisher Scientific, catalog number: K1081)

9. Divergent PCR primer pair (unique to the circRNA)

10. Sodium chloride (NaCl) (Millipore Sigma, catalog number: S9888)

11. MOPS, sodium salt (Na-MOPS) (Research Products International, catalog number: M92040)

12. Trizma^®^ base (Millipore Sigma, catalog number: T1503)

13. EDTA (0.5 M) (Invitrogen, catalog number: AM9260G)

14. Glacial acetic acid (Millipore Sigma, catalog number: A6283)

15. SeaKem^®^ LE agarose (Thermo Fisher Scientific, catalog number: BMA50001)

16. GelRed^®^ nucleic acid gel stain (Biotium, catalog number: 41003)

17. GeneRuler 1 kb Plus DNA ladder (Thermo Fisher Scientific, catalog number: SM1331)

18. TRIzol reagent (Thermo Fisher Scientific, catalog number: 15596026)

19. RNase R (NEB, catalog number: M0100S)

20. RNA Clean and Concentrator-25 (Zymo Research, catalog number: R1017)

21. Nuclease-free water (NEB, catalog number: B1500S)

**Solutions**

1. RNA elution buffer (see Recipes)

2. 10× TAE (see Recipes)

**Recipes**

**1. RNA elution buffer**

**Table:** 

Reagent	Final concentration	Quantity or volume
1 M Na-MOPS (pH 6.0)	10 mM	200 μL
5 M NaCl	300 mM	1200 μL
0.5 M EDTA (pH 8.0)	1 mM	40 μL
Deionized H_2_O	n/a	18.56 mL
Total	n/a	20 mL

**2. 10× TAE**

**Table:** 

Reagent	Final concentration	Quantity or volume
Trizma^®^ base	400 mM	48.5 g
17.4M Glacial acetic acid	200 mM	11.4 mL
0.5 M EDTA (pH 8.0)	10 mM	20 mL
Deionized H_2_O	n/a	Fill up to 1 L
Total		1 L

**Laboratory supplies**

1. Standard pipette tips with a volume capacity of 10 μL, 20 μL, 200 μL, and 1 mL

2. PCR strip tubes (USA Scientific, catalog number: 1402-4708)

3. 1.7 mL tubes (Genesee, catalog number: 24-282)

4. 0.22 μm syringe filters (Millipore Sigma, catalog number: WHA9913-2502)

5. 1 mL disposable syringe (Thermo Fisher Scientific, catalog number: 14-823-30)

6. Tissue culture flask, 75 cm^2^ (VWR, catalog number: 13-680-65)

7. Serological pipettes 10 mL (Genesee, catalog number: 12-104)

## Equipment

1. Pipetman (2, 20, 200, 1,000 μL) (Gilson, catalog number: F167360)

2. Eppendorf^TM^ Centrifuge 5425 (Eppendorf, catalog number: 5405000247)

3. C1000 Touch Thermal Cycler (Bio-Rad Laboratories, catalog number: 1851148)

4. Sub-Cell GT Horizontal Electrophoresis System (Bio-Rad Laboratories, catalog number: 1704403)

5. GelDoc Go (Bio-Rad Laboratories, catalog number: 12009077)

6. Nanodrop 1000 (Thermo Scientific^TM^)

7. Personal microcentrifuge (USA Scientific, catalog number: 2641-0016)

## Software and datasets

1. Benchling (Benchling, Non-Validated Cloud)

This is a free-to-use cloud software. The sequence alignment tool in Benchling was used for the analysis of the Sanger and Nanopore sequencing data.

## Procedure

**A. Synthetic circRNA isolation**

Synthetic circRNAs were generated as described by Du et al. [7] (see General note 1). Skip to section **C** when aiming to detect a synthetic circRNA from a mixture without further purification (see General note 2). Skip to section **B** when verifying a circRNA present in RNA isolated from cells.

1. Agarose gel to isolate circRNA

a. Prepare GelRed^®^-containing 1%–2% agarose gel.

b. Mix the circRNA with 2× RNA loading dye and load at least 100 ng per well to visualize the circRNA.

c. Resolve the RNA at 90 V until the dye reaches three-fourths of the gel.

d. Visualize the gel using a UV transilluminator (GelDoc Go) and cut out the circRNA band.

2. circRNA extraction

a. Crush the gel using a pestle, add 2 volumes (v/w) of RNA elution buffer (e.g., 200 μL of buffer to 100 mg of gel), and incubate at room temperature for at least 30 min or overnight at 4 °C.

b. Use the elution directly as a template for RT-PCR in section C or ethanol precipitate the RNA prior to RT-PCR for long-term storage of the purified circRNA [20]. For ethanol precipitation, remove gel pieces using a 0.22 μm syringe filter.

**B. Total cellular RNA isolation**

The steps below were used to isolate RNA from 80% confluent HEK293T cells grown in a T-75 (75 mm^2^) flask. Skip to section C when starting with isolated RNA.

1. TRIzol extraction

a. Carefully aspirate the media not to disturb the cells.

b. Add 1 mL of TRIzol per 10 cm^2^ of culture flask area, e.g., 7.5 mL for a T-75 (75 cm^2^) culture flask.

c. Gently mix the TRIzol reagent with a serological pipettor until all cells are suspended in the TRIzol reagent (see General note 3).

d. Follow the TRIzol RNA isolation protocol as described by the manufacturer (Thermo Fisher Scientific, MAN0001271). Resuspend the RNA in 50 µL of nuclease-free water for the final step.

e. Measure the concentration of the RNA on a nanodrop (see General note 4).

f. Check your RNA quality by resolving it on a 1% agarose gel. There should be clear bands corresponding to the 28S and 18S ribosomal RNA bands without RNA degradation (see General note 5).

2. RNase R digestion to remove linear RNA

a. Add 1 µL of RNase R to 20 µg of RNA in a 20 μL reaction volume.

b. Incubate at 37 °C for 30 min.

c. Remove the RNase R enzyme using a Zymo RNA Clean and Concentrator-25 column and elute the remaining RNA using 25–50 μL of nuclease-free water.

d. The eluted RNA can be used as a template for section **C**.

**C. uMRT reverse transcription**

The uMRT reverse transcription was adapted from the manufacturer’s protocol (RNAConnect, R1002S).

**Figure 1.**
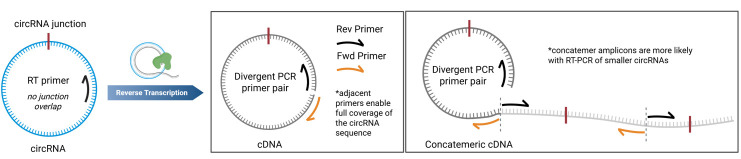
Primer design for RT-PCR of circRNAs. The reverse transcription primer should not overlap with the circRNA junction. The PCR primers must be divergent and should be adjacent for full circRNA coverage. Concatemeric amplicons can be observed from rolling circle reverse transcription.

1. RT primer design

a. The RT primer is designed to be at least 100 nt upstream of the circRNA junction (Figure 1). This ensures that the junction is not found near low-quality sequencing read areas near the termini of the amplicons.

2. Prepare the RNA primer mix

a. In a PCR tube, mix the gene-specific RT primer with the template circRNA and dNTPs (Table 1).

**Table 1.** RNA primer mix

	Concentration	Volume (μL)
RT primer	1 μM	2
circRNA	(1 pg to 1 ug)	1–3
dNTP mix	10 mM each	1
Water (RNase-free)		0–2 (up to 6 µL)
	Total volume	6

b. Incubate the samples in a thermocycler at 95 °C for 30 s, then hold at 4 °C.

3. Prepare uMRT master mix (14 μL/sample) (Table 2).

**Table 2.** uMRT master mix

	Stock concentration	Final concentration*	Volume (µL)
Water (RNase-free)	-	-	1
2× RT reaction buffer	2×	1×	10
UltraMarathonRT	20 U/µL	1 U/μL	1
RNaseOUT^TM^	40 U/µL	2 U/L	1
High boost (R1002S)	-	-	1
		Total	14

*Note: The final concentration is given for a 20 µL volume, as this will be combined with the RNA primer mix created in step C2. High Boost improves cDNA yield from low RNA inputs.

4. Reverse transcription reaction

a. Combine the 2× uMRT master mix (14 µL) with the RNA primer mix (6 µL).

b. Incubate the reverse transcription reaction in a thermocycler under the following settings (Table 3).

**Table 3.** Thermocycler conditions for reverse transcription

Description	Temperature	Time (min)
First-strand synthesis	20 °C*	15
Inactivation	95 °C	1
Hold	4 °C	∞

*Note: The reverse transcription works at 20–42 °C.

**D. PCR amplification**

1. Primer design for PCR

a. The primer design for this method is crucial for being able to sequence whole circRNAs and to prevent amplification of linear RNAs. The primers need to be divergent to ensure the primers only amplify circular RNA, and they need to be adjacent to one another to ensure full coverage of the circRNA sequence (Figure 1), as is discussed in greater detail elsewhere [8,9]. Primers should be at least 100 nt away from the circRNA junction to avoid low-quality read areas near primer binding sites in Sanger sequencing. The same primer can be used for RT and as the reverse primer for the subsequent PCR reaction (see General note 6).

2. Set up the PCR reaction as follows (Table 4):

**Table 4.** PCR reaction setup

Reagent	Concentration	Volume
Water	-	22
DreamTaq Master Mix*	2×	25
cDNA template	1–100 ng/µL	1
Fwd primer	10 µM	1
Rev primer	10 µM	1
	Total	50

*Note: Other PCR setups could be used here instead of DreamTaq.

3. Thermocycler settings (Table 5):

**Table 5.** Thermocycler conditions for PCR

	Temperature (°C)	Time	
Initial denaturing	98	30 s	
Denaturing	98	10 s	30–40 cycles*
Annealing	T_m_-5*	30 s	
Extension	72	1 min*	
Final extension	72	5 min	
	4	hold	

*Note: Adjust the annealing temperature based on the primers melting temperature (T_m_). Above 2 kb, the extension time needs to increase by 1 min/kb. The cycle number can be increased with lower cDNA inputs.

4. Analyzing PCR products by gel electrophoresis

a. Make a 1% agarose gel, stained with GelRed. Adjust the gel percentage based on amplicon size.

b. Load between 2 and 6 µL of PCR product.

5. Visualize the agarose gel

a. Image under UV light or using a GelDoc Go.

**E. Sequencing**

1. PCR cleanup

a. Follow the PCR cleanup protocol as instructed by the DNA Clean and Concentrator-25 kit (Zymo Research, D4033).

b. Measure the concentration of your PCR product on the Nanodrop.

2. Sanger sequencing

a. Sanger sequencing is used for amplicons that are too small for commercial Nanopore sequencing (< 500 bp).

b. Send 15 µL (at least 50 ng/µL) for Sanger sequencing with an appropriate sequencing primer. Instructions may vary based on the vendor used for commercial Sanger sequencing.

3. Nanopore sequencing

a. Use Nanopore sequencing for amplicons greater than 500 bp.

b. Send 10 µL of 20–200 ng/µL for commercial nanopore sequencing. Instructions may vary based on vendor (see General note 7).

## Data analysis

**Sequence alignment of circRNAs in Benchling**

The same data analysis can be done for sequencing files obtained from commercial Sanger and Nanopore sequencing.

1. Create a DNA file as the template for the alignment in Benchling by following the procedure below:

a. Create a new DNA sequence.

b. Set nucleotide type to DNA.

c. Set topology to circular.

d. Paste in the circRNA sequence.

2. Sequence alignment of the circRNA, DNA, and sequencing data

a. Select your circRNA sequence (created in the previous step), select alignments, and upload the sequencing file (FASTA, AB1, or SEQ file).

b. Use the default parameters for the Benchling alignment.

## Validation of protocol

**uMRT produces large circRNA concatemers**

**Figure 2.**
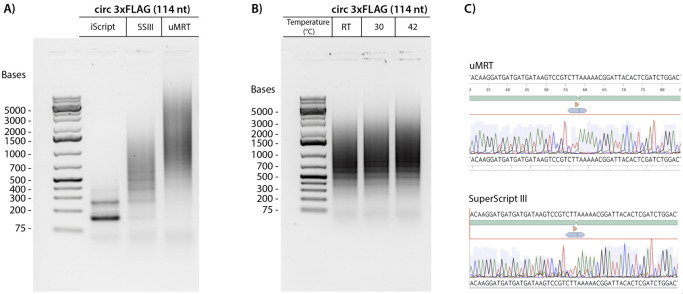
UltraMarathonRT^®^ (uMRT) produces large concatemers of a small circRNA. **(A)** 3xFLAG (114 nt) circular RNA was used as a template for RT-PCR with iScript, SuperScript III (SSIII), and uMRT. **(B)** uMRT performs rolling circle reverse transcription at lower temperatures. 3xFLAG (114 nt) circular RNA was used as a template for RT-PCR with uMRT at room temperature (25 °C), 30 °C, and 42 °C. **(C)** Sanger sequencing results of concatemeric RT-PCR products by SuperScript III and uMRT. The circRNA sequence (top, green annotation) shows the circRNA junction (annotated with the orange triangle).

We initially evaluated the RT-PCR method using a small circRNA encoding a 114 nucleotide (nt) 3xFLAG sequence. The circular 3xFLAG sequence and all primers used in RT-PCR are found in Dataset S1. Details of the synthesis of the 3xFLAG circRNA sequence are provided in a previous publication by Du et al. [7]. We performed reverse transcription of this circRNA using iScript, SuperScript III (SSIII), and uMRT, followed by PCR amplification of the cDNA products with divergent primers (Figure 2A). Less than 2 ng of RNA template (below the Nanodrop detection limit) was used for reverse transcription. 1 µL of the reverse transcription reaction was used as template for the PCR reactions as described in section D. The RT-PCR products from SSIII and uMRT were large concatemers of the circRNA, which was validated by Sanger sequencing (Figure 2C). iScript, which has an active RNase H domain, produced cDNA that was 1–2 times the size of the circRNA, which is under the minimum 150 nt length required for Sanger sequencing with most commercial services. In contrast, SSIII displayed higher processivity, generating a ladder pattern, whereas uMRT showed even greater processivity, producing cDNA concatemers that were 10–20 times the size of the original circRNA (Figure 1A). Despite the presence of multiple PCR products in both SSIII and uMRT samples, these products were suitable for Sanger sequencing, as they represent repeats of the same sequence (Figure 2C, Dataset S1).

We further examined the capability of uMRT to perform rolling circle reverse transcription at lower temperatures. uMRT successfully synthesized cDNA from the 3xFLAG template at room temperature (25 °C), 30 °C, and 42 °C (Figure 2B). Performing reverse transcription at lower temperatures helps minimize RNA degradation and shearing open of circles, which could be advantageous for future applications that involve reverse transcribing low-abundance circular RNAs from cell extracts.

**RT-PCR with larger circRNA sizes**

We tested whether using a processive RT could be useful for detecting larger circRNAs. Specifically, we compared the efficiency of SuperScript III and uMRT in reverse-transcribing the circRNAs of lncATV (420 nt), circ-Znf609 (935 nt), and CVB3-EGFP (1596 nt). The circular RNA sequences and all primers used in RT-PCR are found in Dataset S1. The CVB3-EGFP circRNA was made as described by Du et al. [7], whereas the synthesis of the lncATV and circ-Znf609 circRNAs was done using a different permuted intron exon [21]. The CVB3-EGFP circRNA contains an internal ribosome entry site (IRES), a highly structured RNA element that is commonly added to synthetic circRNAs to enable translation, whereas lncATC and circ-Znf609 are natural RNAs. These circRNA sequences were chosen due to their structured RNA regions and repetitive sequences, which are known to be especially challenging to reverse transcriptases.

**Figure 3.**
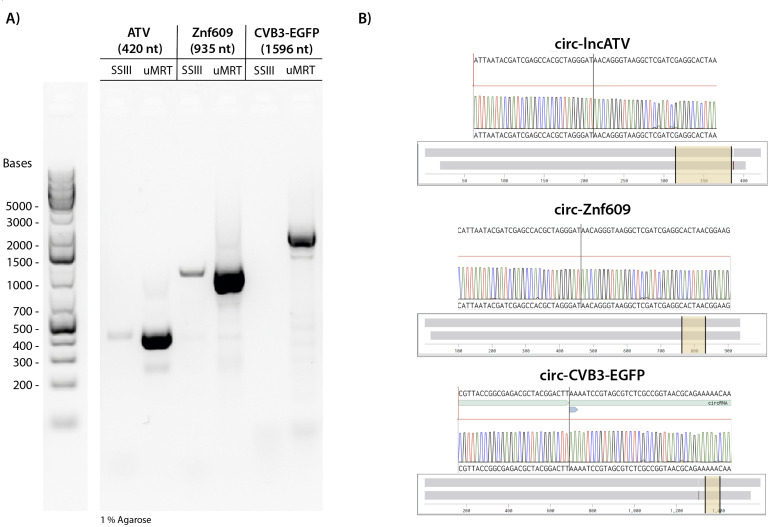
UltraMarathonRT^®^ (uMRT) reverse-transcribes structured and large circRNAs. **(A)** SuperScript III and uMRT were compared in their ability to reverse-transcribe larger circRNAs. The following circRNAs were used as input for the reverse transcription: lncATV (420 nt), circ-Znf609 (935 nt), and CVB3-EGFP (1596 nt). **(B)** Nanopore sequencing results of RT-PCR amplicons. Sequencing results across the junctions are shown (yellow highlighted region). Note that circ-lncATV and circ-Znf609 have identical splicing junctions due to the synthesis method. The whole sequence alignment is shown by the grey bar.

We observed that uMRT successfully reverse-transcribed all circRNAs in our RT-PCR experiments, whereas the RT-PCR using SuperScript III produced only faint amplicons and failed to reverse transcribe the 1596 nt CVB3-EGFP circRNA (Figure 3A). Although SuperScript III is capable of reverse-transcribing large RNA sequences up to 12 kb, the highly structured IRES element in CVB3 likely impedes SuperScript III. We also noted that there are no concatemeric PCR products with these larger circRNAs, which could be due to less rolling circle reverse transcription for the larger circRNAs or PCR bias for the smaller PCR products. We proceeded with Nanopore sequencing following PCR cleanup and found that the amplicons produced by uMRT matched the expected circRNAs (Figure 3B, Dataset S1). In contrast, the amplicons from SuperScript III were not sufficiently concentrated for sequencing. These results indicate that for detecting circular RNA, using a highly processive reverse transcriptase like uMRT is crucial for successful amplification and sequencing.

**Detection of natural circRNAs**

**Figure 4.**
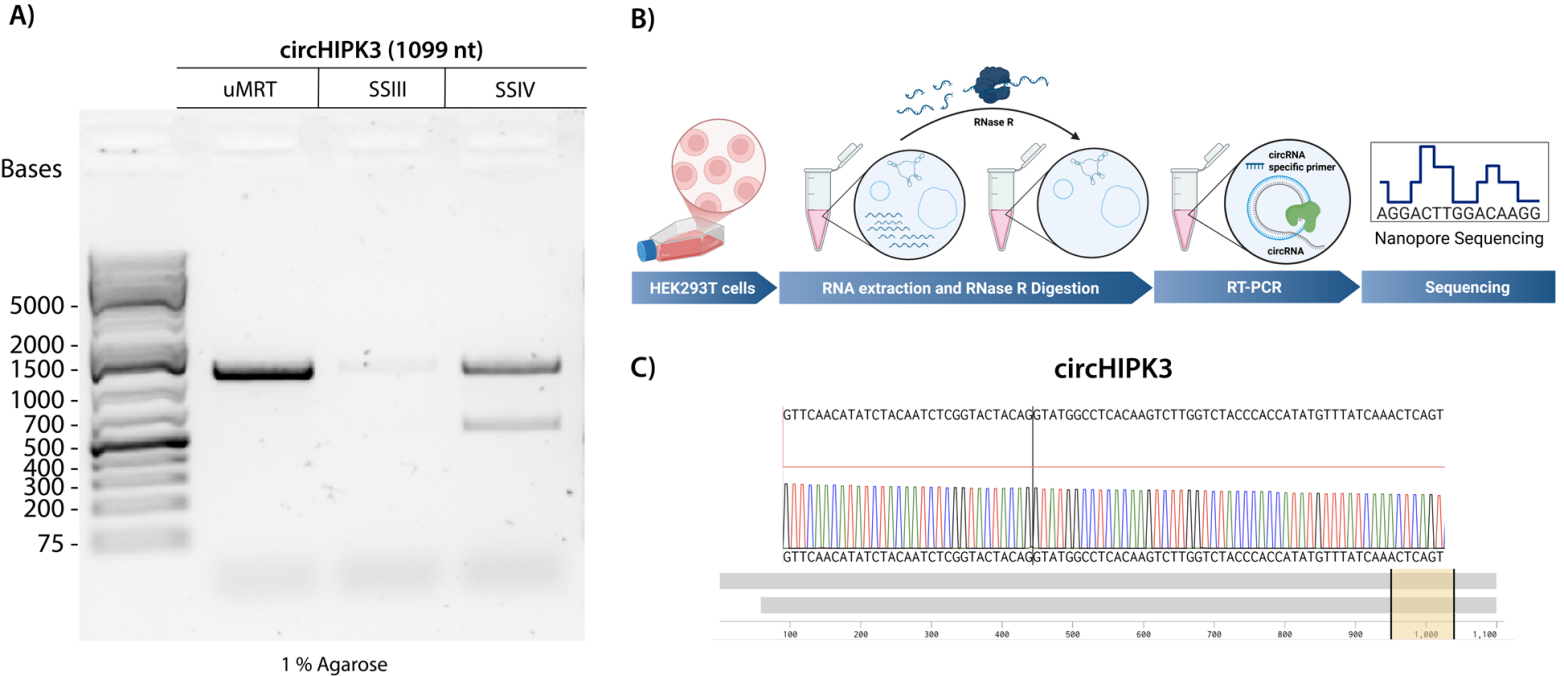
UltraMarathonRT^®^ (uMRT) reverse-transcribes natural circRNAs efficiently. **(A)** RT-PCR of circHIPK3 using uMRT, SuperScript III (SSIII), and SuperScript IV (SSIV). The expected RT-PCR product for circHIPK3 is 1,099 nt. **(B)** Method overview for detecting natural circRNAs. RNA extraction from HEK293T cells is treated with RNase R to remove linear RNA. **(C)** Nanopore sequencing results of the uMRT RT-PCR amplicon. Sequencing results across the junction are shown (yellow highlighted region). The whole sequence alignment is shown by the grey bars.

We then tested if using a highly processive RT that operates at lower temperatures improves the detection of natural circRNAs. We investigated whether RT-PCR with uMRT could effectively detect the natural circRNA circHIPK3 (1,099 nt) that is known to be present in HEK293T cells [22]. RNA was isolated from confluent HEK293T cells using Trizol (per Invitrogen user guide), treated with RNase R to remove linear RNAs as described in section B, and then utilized as a template for RT-PCR (Figure 4B). The resultant cDNA was amplified using divergent primers and sequenced using commercial nanopore sequencing by Plasmidsaurus.

RT-PCR with uMRT resulted in the highest intensity amplicon, whereas that with SuperScript III produced a single faint amplicon, and SuperScript IV produced an additional nonspecific band (Figure 4A). The band produced by uMRT matched the expected sequence of circHIPK3, spanning the back-splicing junction (Figure 4C). In contrast, the SuperScript III amplicon was too diluted for sequencing, and the SuperScript IV produced two amplicons.

## General notes and troubleshooting

**General notes**

1. For step 1 of the uMRT reverse transcription, the circRNAs used in this protocol were produced using the TRIC intron system described by Du et al. [7] or a permuted intron exon system [21]. The circRNA template concentrations were below 2 ng/µL (below the Nanodrop detection limit).

2. When sequencing synthetic circRNAs, you can start with an impure sample or gel-extract the circRNA band to verify the identity of a particular band.

3. To check if the cell debris has been suspended in Trizol, you can look at the cells under a brightfield microscope. If you move the culture flask under the microscope, you should see the cell debris move in the TRIzol.

4. The A260/280 ratio is around 2.0 for pure RNA. A yield of 100 µg of total cell RNA is expected from 80% confluent HEK293T cells in a T-75 tissue culture flask.

5. RNA quality can be checked using a TapeStation or BioAnalyzer instead of an agarose gel.

6. Proper primer design is essential to avoid seeing false positives. When designing RT-PCR primers, make sure the primers do not include the circRNA junction. The amplicon that is produced should span the junction, but the primers should not include it. Including the circRNA junction in the primer can lead to amplification from linear templates. For more information on primer design for circRNAs, see Panda and Gorospe [8].

7. When sequencing with Plasmidsaurus, no primers are needed for nanopore sequencing. Note that 25 nt of the 5′ and 3′ termini of the amplicons may not be fully resolved in Nanopore sequencing.

**Troubleshooting**

Problem 1: Sanger sequencing fails to span the circRNA ligation site.

Possible cause: Long repeats of A or T or tandem repeats.

Solution: If your circRNA contains long repeats of A or T, then standard Sanger sequencing may be unable to sequence the amplicons. In this case, it may be best to send the amplicons for nanopore sequencing, but your amplicons must meet the length required for Nanopore sequencing (>500 bp for commercial Nanopore sequencing through Plasmidsaurus). If the amplicons are too short for commercial Nanopore sequencing, the PCR product can be cloned into a vector and sent for whole plasmid sequencing.

Problem 2: PCR products do not match the expected size.

Possible cause: Primers have alternative binding sites, or the polymerase is not processive enough.

Solution: Check if your primers have potential alternative binding sites, as this might produce different products. Design another set of primers and check for alternative binding sites. We found that a melting temperature of around 60 °C works well for primers used with the DreamTaq polymerase.

Problem 3: circRNA does not contain good binding sites for the divergent primers.

Possible cause: Some circRNAs contain highly structured elements with lots of repeats. This can make it challenging to design divergent primers that amplify the entire circRNA.

Solution: It may be necessary to offset your primers by a few base pairs if there are no good binding sites for creating two divergent primers right next to one another. This can result in lower-quality results from Sanger sequencing, as there are now concatemers with slightly different repeats. This is not an issue for Nanopore sequencing.

Problem 4: Failed PCR amplification from RT-PCR of large circRNAs (>2 kb).

Possible cause: DreamTaq may not be processive or high-fidelity enough to amplify the cDNA.

Solution: Use a high-fidelity polymerase, such as Q5 High-Fidelity polymerase.

## Supplementary information

The following supporting information can be downloaded here:

1. Dataset S1. circRNA sequences and primers used in RT-PCR alongside corresponding sequencing data.

## References

[1] Suzuki H. (2006). Characterization of RNase R-digested cellular RNA source that consists of lariat and circular RNAs from pre-mRNA splicing. Nucleic Acids Res. 34(8): e63–e63. 10.1093/nar/gkl151 PMC1458517 16682442

[2] Chen L. L. (2020). The expanding regulatory mechanisms and cellular functions of circular RNAs. Nat Rev Mol Cell Biol. 21(8): 475 490 490. 10.1038/s41580-020-0243-y 32366901

[3] Kristensen L. S., Jakobsen T., Hager H. and Kjems J. (2021). The emerging roles of circRNAs in cancer and oncology. Nat Rev Clin Oncol. 19(3): 188 206 206. 10.1038/s41571-021-00585-y 34912049

[4] Dong X., Bai Y., Liao Z., Gritsch D., Liu X., Wang T., Borges-Monroy R., Ehrlich A., Serrano G. E., Feany M. B., .(2023). Circular RNAs in the human brain are tailored to neuron identity and neuropsychiatric disease. Nat Commun. 14(1): e1038/s41467–023–40348–0. 10.1038/s41467-023-40348-0 PMC10507039 37723137

[5] Verduci L., Tarcitano E., Strano S., Yarden Y. and Blandino G. (2021). CircRNAs: role in human diseases and potential use as biomarkers. Cell Death Dis. 12(5): e1038/s41419–021–03743–3. 10.1038/s41419-021-03743-3 PMC8113373 33976116

[6] Wesselhoeft R. A., Kowalski P. S. and Anderson D. G. (2018). Engineering circular RNA for potent and stable translation in eukaryotic cells. Nat Commun. 9(1): e1038/s41467–018–05096–6. 10.1038/s41467-018-05096-6 PMC6035260 29980667

[7] Du Y., Zuber P. K., Xiao H., Li X., Gordiyenko Y. and Ramakrishnan V. (2024). Efficient circular RNA synthesis for potent rolling circle translation. Nat Biomed Eng. 9(7): 1062 1074 1074. 10.1038/s41551-024-01306-3 39672985 PMC12270912

[8] Panda A. and Gorospe M. (2018). Detection and Analysis of Circular RNAs by RT-PCR. Bio Protoc. 8(6): e2775. 10.21769/bioprotoc.2775 PMC5891140 29644261

[9] Dodbele S., Mutlu N. and Wilusz J. E. (2021). Best practices to ensure robust investigation of circular RNAs: pitfalls and tips. EMBO Rep. 22(3): e202052072. 10.15252/embr.202052072 PMC7926241 33629517

[10] Nielsen, A. F., Bindereif, A., Bozzoni, I., Hanan, M., Hansen, T. B., Irimia, M., Kadener, S., Kristensen, L. S., Legnini, I., Morlando, M. et al.(2022). Best practice standards for circRNA research. Nat Methods. 19(10): 1208 10.1038/s41592-022-01487-2 35618955 PMC9759028

[11] Su C. I., Chuang Z. S., Shie C. T., Wang H. I., Kao Y. T. and Yu C. Y. (2024). A cis-acting ligase ribozyme generates circular RNA in vitro for ectopic protein functioning. Nat Commun. 15(1): 6607 10.1038/s41467-024-51044-y 39098891 PMC11298514

[12] Liao K. C., Eshaghi M., Hong Z., Saw T. Y., Lim J. A. J., Han J., Aw J. G. A., Tan K. Y., Yap A., Gao X., .(2025). Characterization of group I introns in generating circular RNAs as vaccines. Nucleic Acids Res. 53(4): e1093/nar/gkaf089. 10.1093/nar/gkaf089 PMC11879131 40036878

[13] Liu Z., Tao C., Li S., Du M., Bai Y., Hu X., Li Y., Chen J. and Yang E. (2021). circFL-seq reveals full-length circular RNAs with rolling circular reverse transcription and nanopore sequencing. eLife. 10: e69457. https://doi.org/10.7554/elife.69457 PMC8550772 34647522

[14] Das A., Das D. and Panda A. C. (2021). Validation of Circular RNAs by PCR. Methods Mol Biol. 2392: 103 114 114. 10.1007/978-1-0716-1799-1_8 34773618

[15] Malik O., Khamis H., Rudnizky S., Marx A. and Kaplan A. (2017). Pausing kinetics dominates strand-displacement polymerization by reverse transcriptase. Nucleic Acids Res. 45(17): 10190 10205 10205. 10.1093/nar/gkx720 28973474 PMC5737391

[16] Unlu I., Maguire S., Guan S. and Sun Z. (2024). Induro-RT mediated circRNA-sequencing(IMCR-seq) enables comprehensive profiling of full-length and long circular RNAs from low input total RNA. Nucleic Acids Res. 52(13): e55–e55. 10.1093/nar/gkae465 PMC11260445 38850158

[17] Guo L. T., Adams R. L., Wan H., Huston N. C., Potapova O., Olson S., Gallardo C. M., Graveley B. R., Torbett B. E., Pyle A. M., .(2020). Sequencing and Structure Probing of Long RNAs Using MarathonRT: A Next-Generation Reverse Transcriptase. J Mol Biol. 432(10): 3338 3352 3352. 10.1016/j.jmb .2020.03.022 32259542 PMC7556701

[18] Zhao C., Liu F. and Pyle A. M. (2017). An ultraprocessive, accurate reverse transcriptase encoded by a metazoan group II intron. RNA. 24(2): 183 195 195. 10.1261/rna.063479 .117 29109157 PMC5769746

[19] Chu Y., Wang T., Dodd D., Xie Y., Janowski B. A. and Corey D. R. (2015). Intramolecular circularization increases efficiency of RNA sequencing and enables CLIP-Seq of nuclear RNA from human cells. Nucleic Acids Res. 43(11): e75–e75. 10.1093/nar/gkv213 PMC4477644 25813040

[20] Green M. R. and Sambrook J. (2020). Precipitation of RNA with Ethanol. Cold Spring Harb Protoc. 2020(3): pdb.prot101717. https://doi.org/10.1101/pdb.prot101717 32123016

[21] Puttaraju M. and Been M. (1992). Group I permuted intron-exon(PIE) sequences self-splice to produce circular exons. Nucleic Acids Res. 20(20): 5357 5364 5364. 10.1093/nar/20.20.5357 1279519 PMC334342

[22] Zheng Q., Bao C., Guo W., Li S., Chen J., Chen B., Luo Y., Lyu D., Li Y., Shi G., .(2016). Circular RNA profiling reveals an abundant circHIPK3 that regulates cell growth by sponging multiple miRNAs. Nat Commun. 7(1): 11215 10.1038/ncomms11215 27050392 PMC4823868

